# Living with 20 Medications, Attempting Suicide with 15: A Critical Perspective on the Healthcare System through a Somatization Disorder Case Report

**DOI:** 10.1192/j.eurpsy.2025.1018

**Published:** 2025-08-26

**Authors:** M. E. Çelik, F. Ekici

**Affiliations:** 1 Psychiatry, Selcuk University, Konya, Türkiye

## Abstract

**Introduction:**

Patients with somatization disorder frequently seek medical evaluations for unexplained symptoms, strongly believing they are physically ill and often rejecting psychosocial explanations. In Turkey, easy and low-cost access to healthcare and medications via the General Health Insurance system encourages frequent hospital visits. High patient loads and short consultation times hinder thorough assessments, complicating diagnoses like somatization disorder. Consequently, physicians may practice “defensive medicine,” over-ordering tests and medications to minimize risks. These practices reinforce patients’ beliefs in having an organic illness and increase the risk of polypharmacy.

**Objectives:**

This case discusses a patient with somatization disorder experiencing multiple hospital admissions and polypharmacy due to the dynamics of the Turkish healthcare system.

**Methods:**

A 31-year-old woman was admitted after a suicide attempt, exhibiting depressive symptoms and psychosomatic complaints. Detailed examinations of her socio-demographic data, medical and psychiatric history, current complaints, medication use, and past hospitalizations were conducted.

**Results:**

Since age 16, the patient frequently presented with fainting, convulsions, nausea, and vomiting, undergoing extensive evaluations. She repeatedly visited emergency services for chest pain radiating to her left arm and jaw; coronary angiography found no cardiac pathology. Despite no organic cause, she was prescribed 15 different medications by various specialties, reaching 20 tablets daily, covering cardiovascular, gastrointestinal, endocrine, and respiratory systems. Due to family issues, divorce processes, and social stressors, she exhibited depressive and psychosomatic symptoms, attempted suicide 7 times, and was hospitalized in psychiatric wards 12 times. In her latest attempt, she ingested 15 fluoxetine tablets. Psychiatric evaluation revealed ongoing somatic complaints, and polypharmacy was adversely affecting her health. After consultations, unnecessary non-psychiatric medications were discontinued. Her treatment was adjusted to venlafaxine 300 mg/day, clozapine 50 mg/day, and mirtazapine 30 mg/day.

**Conclusions:**

This case illustrates how structural issues in the healthcare system adversely affect patients with somatization disorder, increasing polypharmacy risk. Unnecessary tests and treatments reinforce beliefs in organic illness and complicate management. Healthcare professionals should approach such patients carefully, avoid defensive medicine practices, and consider early psychiatric evaluation. Policy-level changes are needed for the healthcare system to better address these patients’ needs.

**Disclosure of Interest:**

None Declared

